# Optimization of the fermentation process of *Cordyceps sobolifera* Se-CEPS and its anti-tumor activity in vivo

**DOI:** 10.1186/s13036-016-0029-0

**Published:** 2016-06-23

**Authors:** Shengli Yang, Hui Zhang

**Affiliations:** The College of Pharmaceutical Science, Zhejiang University of Technology, Hangzhou, 310014 People’s Republic of China; Zhejiang Institute of Quality Inspection Science, Hangzhou, 310013 People’s Republic of China

**Keywords:** *Cordyceps sobolifera*, Extracellular polysaccharide, Anti-tumor, Fermentation

## Abstract

**Background:**

*Cordyceps sobolifera* (*C. sobolifera*) isolated from cicadae was used as the starting fungus to produce selenium-enriched *C. sobolifera* extracellular polysaccharide (Se-CEPS). An orthogonal experimental design based on a single-factor experiment was used to optimize the *C. sobolifera* fermentation conditions, including the potato juice, peptone, and KH_2_PO_4_ concentrations. Ultraviolet (UV) and infrared (IR) analyses of CEPS and Se-CEPS were conducted, as well as an in vivo anti-tumor analysis.

**Results:**

Under optimal conditions (i.e., 40 potato juice, 0.4 KH_2_PO_4_, and 0.5 % peptone), the fermentation yield of Se-CEPS was 5.64 g/L. UV and IR spectra showed that Se-CEPS contained a characteristic absorption peak of a selenite Se = O double bond, demonstrating the successful preparation of Se-CEPS. Activity tests showed that Se-CEPS improved the immune organ index, serum cytokine content, and CD8^+^ and CD4^+^ T lymphocyte ratio in colon cancer CT26 tumor-bearing mice, thereby inhibiting tumor growth. When combined with 5-FU, Se-CEPS reduced the toxicity and enhanced the function of 5-FU.

**Conclusion:**

The result of these experiments indicated that orthogonal experimental design is a promising method for the optimization of Se-CEPS production, and the Se-CEPS from *C. sobolifera* can improve the anti-tumor capacity of mice.

**Electronic supplementary material:**

The online version of this article (doi:10.1186/s13036-016-0029-0) contains supplementary material, which is available to authorized users.

## Background

*Cordyceps sobolifera* is a rare and unique medicinal fungus that exhibits characteristics of both animals and plants. *C. sobolifera*, a well-known and valued traditional Chinese medicine, is an entomogenous fungal species that is parasitic on wing-less cicada nymphs [1]. Modern medical research and applications have shown that *C. sobolifera* exhibits various functions such as enhancing immunity [2, 3], having anti-aging and anti-fatigue activities [4], having anti-tumor activity [4], improving renal function [5–8], and providing nourishment and strength [4]. *C. sobolifera* and *Cordyceps sinensis* belong to the same insect fungi complex and contain a similar active ingredient; *C. sinensis* has been widely harvested, and the natural supplies have been markedly depleted. Therefore, *C. sobolifera* is often regarded as a substitute for *C. sinensis*. The growth of *C. sobolifera* requires a specific ecological environment and host insects. Moreover, the harvesting of *C. sobolifera* has also become extensive, leading to a steady decline of available sources. Therefore, the use of artificially cultivated *C. sobolifera* mycelium to replace natural *C. sobolifera* has emerged as a future option for its development. In recent years, there have been extensive investigations and reports on *C. sinensis*, but reports on *C. sobolifera* are rare. Because *C. sobolifera* and *C. sinensis* have similar chemical compositions, the medicinal value of the two species is similar, providing the theoretical basis for the substitution of *C. sinensis* with *C. sobolifera* [9–11].

Selenium is an essential trace element that is necessary for maintaining the normal physiological metabolism of the human body [12]. Most diseases of the human body, such as anemia, coronary heart disease, diabetes, and cancer, are related to a lack of selenium [13, 14]. Research has shown that organic selenium is more effective and safer than inorganic selenium as a dietary supplement [15] and that the biological activity of selenium polysaccharide is markedly higher than that of selenium or polysaccharide alone [16]. Selenium polysaccharide is an organic selenium compound composed of selenium and biological polysaccharide, and it exhibits numerous biological effects, such as antioxidation, anti-tumor, immunity enhancement, and blood lipid reduction activities [17, 18].

## Results and discussion

### Orthogonal test for optimization of *C. sobolifera* extracellular polysaccharide (Se-CEPS) fermentation conditions

The effects of different fermentation culture compositions and concentrations on extracellular polysaccharide production were studied. On the basis of a single factor test (Additional file 1: Figures S1, S2, S3, S4, S5 and S6) , three factors were selected: the potato juice, peptone, and KH_2_PO_4_ concentrations, and an L_9_(3^3^) orthogonal test was conducted. Based on the known literature and previous experiments, Se-CEPS was taken as the evaluation index and used to optimize the submerged fermentation conditions. In accordance with the design of the orthogonal experiment shown in Table 1, the effects of different fermentation culture compositions on *C. sobolifera* mycelium secreted extracellular polysaccharide were investigated, and the results are shown in Table 2. Based on the results, the experimental program for optimization was A_2_B_2_C_2_.

**Table 1:** Factors and levels of fermentation condition

Levels	Parameters		
	Potato juice (%) A	Peptone (%) B	KH_2_PO_4_ (%) C
1	20	0.2	0.2
2	40	0.5	0.4
3	60	1.0	0.6

**Table 2:** Results and analysis of orthogonal experiment design in submerged fermentation conditions of *C. sobolifera*

No.	Factors			Extracellular polysaccharide(g/L)
	A	B	C	
1	1	1	1	3.48 ± 0.27
2	1	2	2	5.22 ± 0.39
3	1	3	3	4.35 ± 0.41
4	2	1	2	5.17 ± 0.38
5	2	2	3	5.61 ± 0.40
6	2	3	1	4.73 ± 0.26
7	3	1	3	3.64 ± 0.22
8	3	2	1	4.97 ± 0.29
9	3	3	2	4.23 ± 0.21
T_1_	13.05	12.29	13.18	41.40(T)
T_2_	15.51	15.80	14.62	
T_3_	12.84	13.31	13.60	
k_1_	4.35	4.10	4.39	
k_2_	5.17	5.27	4.87	
k_3_	4.28	4.44	4.53	
r	0.82	1.17	0.48	

From the variance analysis (Table 3), the order of influence of each factor on *C. sobolifera* mycelium extracellular polysaccharide production was B (peptones) > A (potato) > C (KH_2_PO_4_). The three factors significantly affected the results. Ultimately, the optimum conditions for producing *C. sobolifera* mycelium extracellular polysaccharide were determined as A_2_B_2_C_2_, that is, potato juice, 40; KH_2_PO_4_, 0.4; and peptone, 0.5 %. Under these conditions, a maximum Se-CEPS production amount of 5.64 g/L was obtained, and the organic Se content in Se-CEPS was 1.9 mg∙kg^−1^.

**Table 3:** Analysis of variance

Source	SS	Df	MS	F	F_0.05(2,2)_
Potato juice (A)	1.4694	2	0.7347	58.72901679	*
Peptone (B)	2.1734	2	1.0867	86.86650679	*
KH_2_PO_4_ (C)	0.3656	2	0.1828	14.61231015	*
Error (e)	0.2502	20	0.01251		
Total	4.2586	27			

*:F_0.05_ (2,2) = 19.0, F_0.01_ (2,2) = 99.0。

### UV spectra of sodium selenite and polysaccharides

**Fig. 1:**
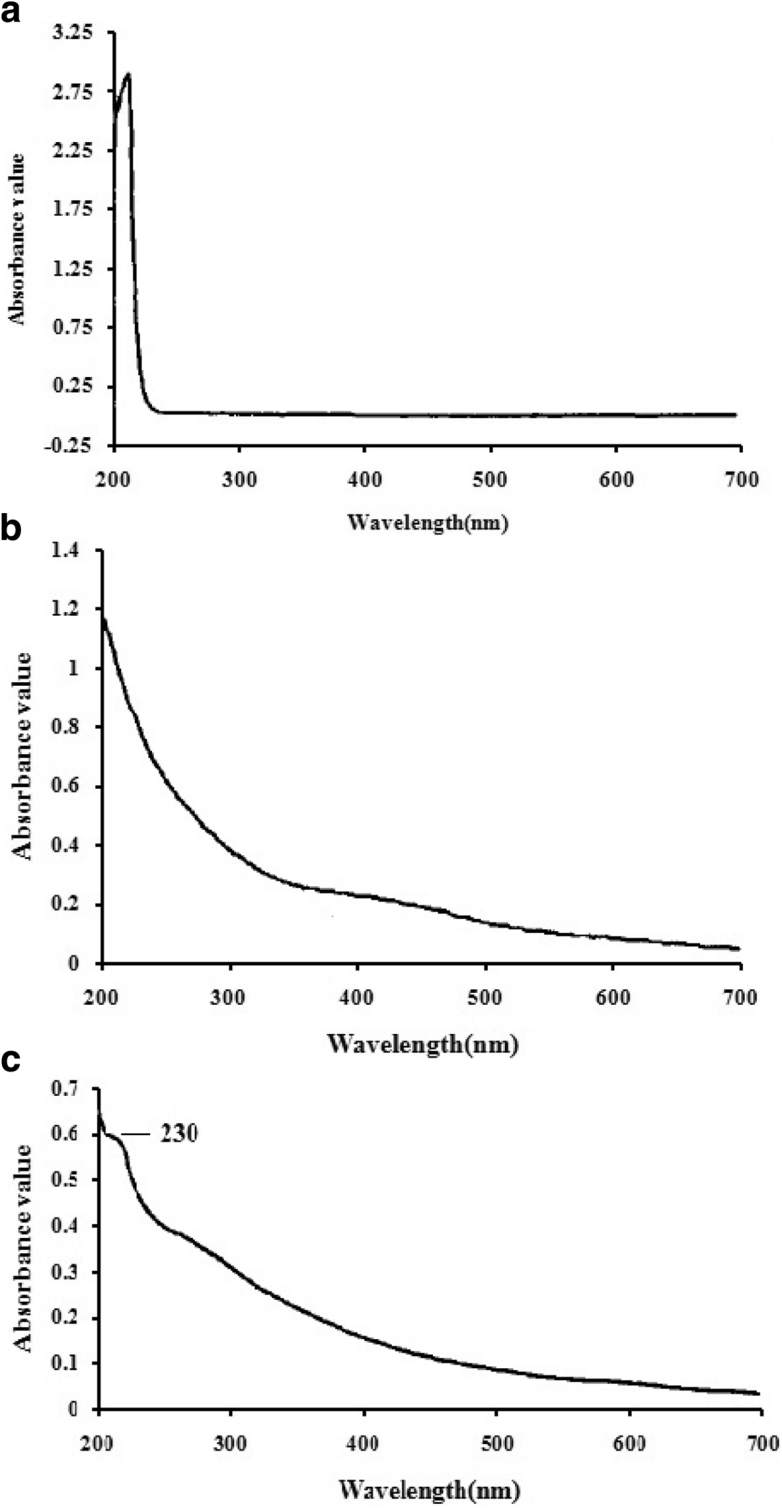
UV spectra of sodium selenite (**a**), CEPS (**b**) and Se-CEPS (**c**) in the range of 200–700 nm

UV spectra of sodium selenite and polysaccharide samples were recorded on a TU-1900 spectrophotometer. As shown in Fig. 1a, the UV spectrum of sodium selenite showed a peak at 220 nm. The UV spectrum of Se-CEPS showed a peak at 230 nm (Fig. 1b, c), which was absent in the spectrum of CEPS. There were apparent differences among the Se-CEPS spectra, indicating that selenium might cause significant chemical modifications in polysaccharides. The Se-CEPS and CEPS fractions had no absorption peak at 260 or 280 nm in their UV spectra, indicating the absence of nucleic acid and protein.

### The infrared spectra of CEPS and Se-CEPS

**Fig. 2:**
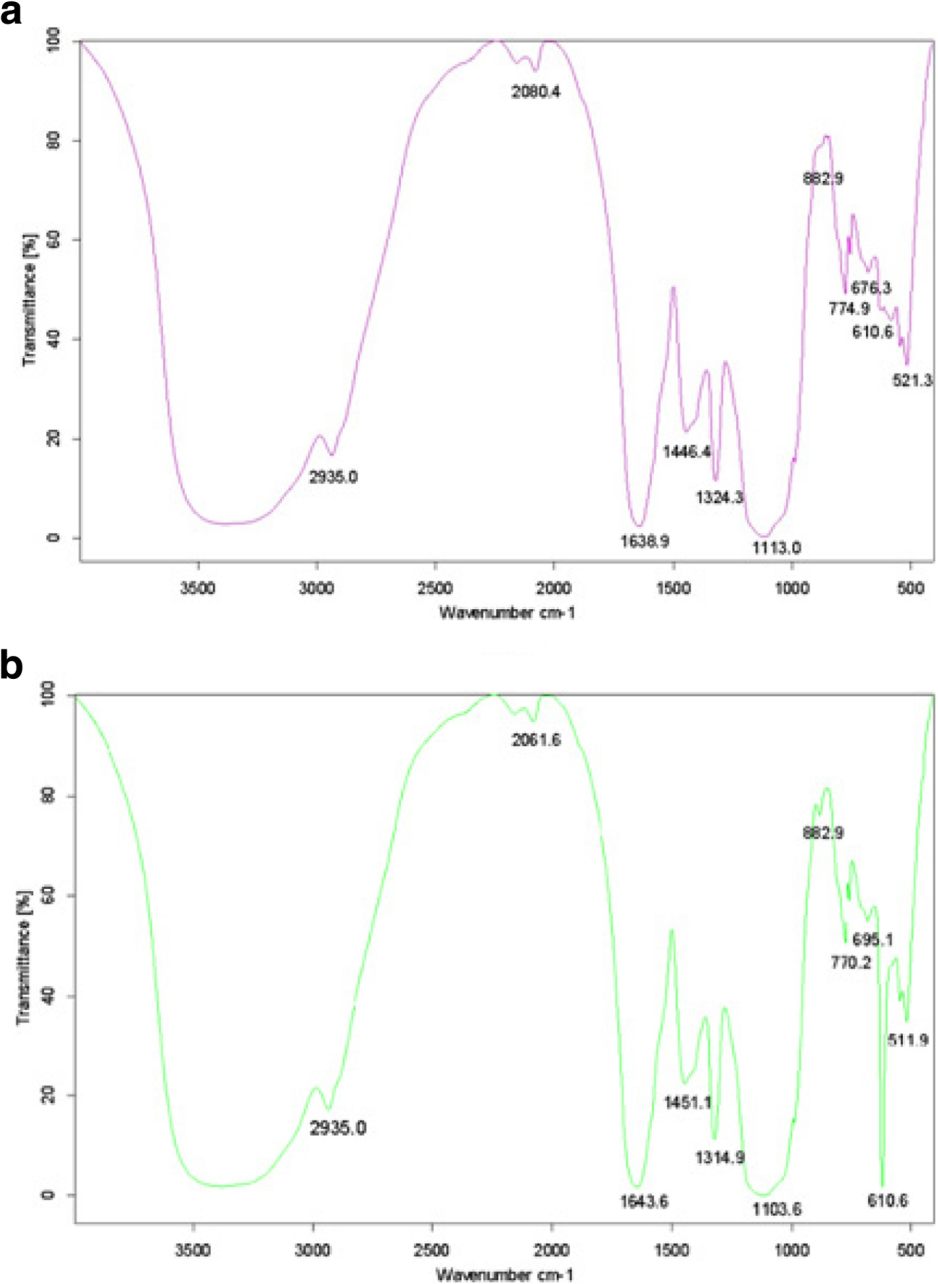
IR spectra of the CEPS (**a**) and Se-CEPS (**b**) in the range of 400–4000 nm

The FT-IR spectrum of Se-CEPS (Fig. 2) showed a strong band in the range of 3200–3600 cm^−1^, which was attributed to the stretching vibration of O–H in the constituent sugar residues. The band at 2935.0 cm^−1^ was associated with the stretching vibration of C–H in the sugar ring, and the bands in the region of 1643.6 cm^−1^ were due to associated water. Absorption bands for the polysaccharides in the range of 950–1200 cm^−1^ were found in cases in which C–O–C and C–O–H link band positions were found. Absent in the spectrogram of CEPS, a weak characteristic absorption band in that of Se-CEPS was found at 882.9 cm^−1^, indicating an asymmetrical Se = O stretching vibration of selenium ester [19], and another characteristic absorption band was found at 610.6 cm^−1^, indicating an asymmetrical Se − O − C stretching vibration [20], which demonstrated that Se-CEPS was successfully modified by selenylation.

### Effects of Se-CEPS on tumor growth, immune organ index, and body weight in tumor-bearing mice

To determine whether Se-CEPS can inhibit tumor growth in vivo, a CT26 colon cancer tumor-bearing mouse model was constructed. The intragastric administration of Se-CEPS significantly inhibited the growth of CT26 tumors, and the effect was dose dependent. At a dose of 200 mg/kg, the tumor suppression rate reached 52.24 %, which was significantly different compared to the control, indicating that Se-CEPS exhibits a strong antitumor activity in vivo (Table 4). The thymus and spleen are important immune organs and are the main locations where immune cell differentiation, maturation, and settlement occur. Moreover, these organs are important locations for immune cells to make contact with antigens during the immune response. The values of the spleen index and thymus index can reflect the strength of the nonspecific immune function. The results showed that compared with the control group, the spleen index and thymus index values of tumor-bearing mice in the three Se-CEPS dose groups, namely, high (200 mg/kg), middle (100 mg/kg), and low (50 mg/kg), significantly increased. Compared with the control group, the parameters of each CEPS dose group also increased but were significantly lower than those of the Se-CSPS groups at the same dose. Compared with the control group, the spleen index, thymus index, and body weight of the 5-FU group were significantly decreased. 5-FU was found to exhibit strong toxicity (Table 4 and Table 5), killing the cancer cells while damaging the immune system. The 200 mg/kg Se-CEPS dose combined with 5-FU improved the anti-tumor effect of 5-FU and improved the immune organ index and weight loss caused by 5-FU. The hair of the mice in the 5-FU group became dull and scattered, and their stool was loose. Some animals in this group died during the administration of the drug. The hair of the mice in the Se-CEPS + 5-FU combined drug group became smooth and shiny, and this effect occurred quickly. Moreover, their stool was normal. These results indicate that Se-CEPS exhibits good in vivo anti-tumor activity and, when combined with 5-FU, reduces the toxicity and increases the efficiency of 5-FU.

**Table 4:** Effects of Se-CEPS on the body weight, tumor growth in CT26-bearing mice (*n* = 8, *p < 0.05 vs model group)

Group	Dosage(mg/kg)	Body weight(g)	Tumor weight(g)	Inhibitory rate(%)
Model control	—	20.34 ± 2.13	1.34 ± 0.31	—
5-FU group	20	16.21 ± 1.86*	0.47 ± 0.16*	64.93
CEPS group	50	20.27 ± 0.98	1.27 ± 0.28	5.22
	100	20.04 ± 2.01	1.03 ± 0.19	23.13
	200	20.13 ± 1.46	0.91 ± 0.33	32.09
Se-CEPS group	50	20.72 ± 2.14	1.06 ± 0.25	20.90
	100	20.41 ± 0.79	0.82 ± 0.14	38.81
	200	20.42 ± 1.68	0.64 ± 0.09*	52.24
Se-CEPS + 5-FU group	200(Se-CEPS) + 20(5-FU)	18.15 ± 2.17*	0.36 ± 0.17*	73.13

**Table 5:** Effects of Se-CEPS on immune organ index in CT26-bearing mice (*n* = 8, *p < 0.05 vs model group, #*p* < 0.05 vs 5-FU group)

Group	Dosage (mg/kg)	Spleen index (mg/g)	Thymus index (mg/g)
Model control		9.18 ± 0.74	4.12 ± 0.29
5-FU group	20	5.34 ± 0.52	2.51 ± 0.27
CEPS group	50	9.22 ± 0.49	4.52 ± 0.44
	100	9.28 ± 0.98	4.78 ± 0.31
	200	9.45 ± 0.72	5.11 ± 0.37
Se-CEPS group	50	9.41 ± 0.63	5.14 ± 0.42
	100	10.18 ± 0.47	5.58 ± 0.28*
	200	10.92 ± 0.32*	5.97 ± 0.31*
Se-CEPS + 5-FU group	200(Se-CEPS) + 20(5-FU)	8.04 ± 0.49*#	3.73 ± 0.25*#

### Effect of Se-CEPS on the content of cytokines in the serum of tumor-bearing mice

**Fig. 3:**
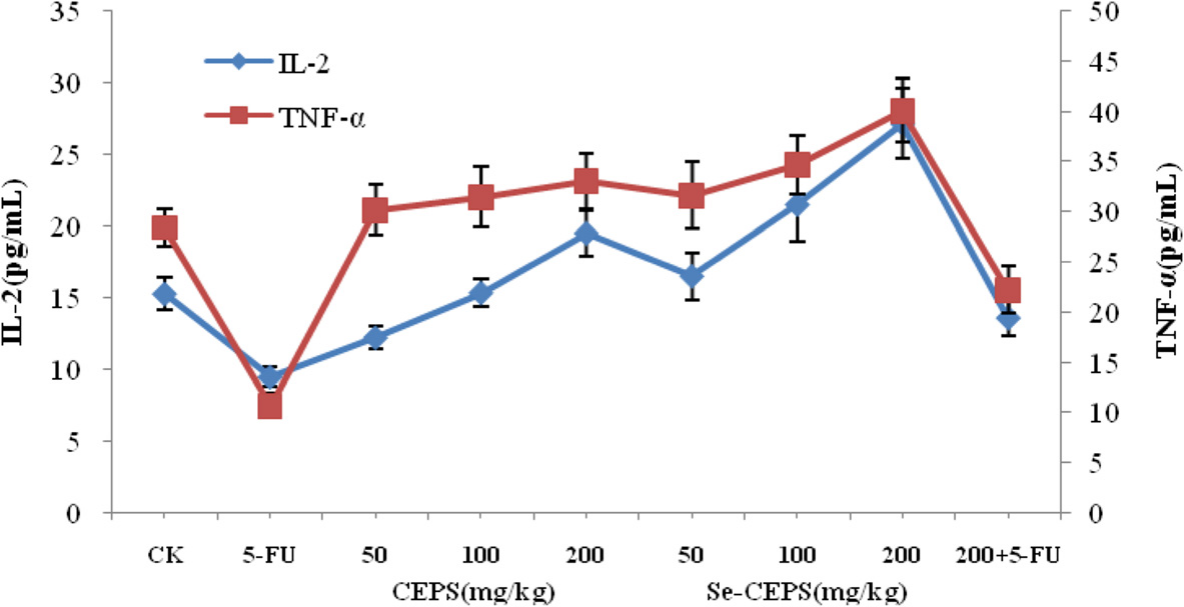
Effects of Se-CEPS on the levels of IL-2 and TNF-α in serum of CT26-bearing mice

In this study, the ELISA method was used to measure the levels of TNF-α and IL-2 in the serum of CT26 colon cancer tumor-bearing mice. As shown in Fig. 3, compared with the control group, the TNF-α and IL-2 contents in the serum of tumor-bearing mice in the high Se-CEPS dose group significantly increased (*p* < 0.05). The cytokine content in the serum of mice in the positive control group was lower than that in the control group. Each dose group of CEPS showed the same trend as Se-CEPS, but the cytokine levels were lower than those in the Se-CEPS groups. The levels of IL-2 and TNF-α in the 5-FU + Se-CEPS combination group were higher than those in the 5-FU group.

### Effects of polysaccharides on CD8^+^ and CD4^+^ T cell counts in the spleen of tumor-bearing mice

CD8^+^ and CD4^+^ T lymphocytes are important effector cells that directly kill tumor cells in vivo. Flow cytometry was used to measure the proportions of CD8^+^ and CD4^+^ T lymphocytes in the spleen. The results showed that treatment with different doses of Se-CEPS increased the proportions of CD8^+^ and CD4^+^ T lymphocytes in mice to varying degrees. A significant difference was observed between the 200 mg/kg dose group and the control group (Table 6). The proportion of CD8^+^ T lymphocytes in the 5-FU group decreased, but this change was not significantly different compared with the control group. The proportion of CD8^+^ T lymphocytes in the Se-CEPS + 5-FU combination group was higher than that in the 5-FU group. As shown in Table 6, there was no significant difference (*p* > 0.05) in the CD8^+^T cell counts between the CEPS and Se-CEPS groups. The CD4^+^T cell counts in the 5-FU group were markedly lower than those in the control group. Compared with the 5-FU group, the Se-CEPS and CEPS treatments enhanced the of CD4^+^T counts (*p* < 0.05). In addition, the CD4^+^/CD8^+^T cell ratios in the Se-CEPS groups were markedly higher compared with the 5-FU group (Table 6). Moreover, Se-CEPS + 5-FU administration demonstrated stronger effects on the counts of CD4^+^T cells and the CD4^+^/CD8^+^T cell ratio compared with the Se-CEPS or 5-FU treatment alone.

**Table 6:** Effects of Se-CEPS on the percentage of CD8^+^ and CD4^+^ T cells among total T lymphocytes in CT26-bearing mice (*n* = 8, *p < 0.05 vs model group, #*p* < 0.05 vs 5-FU group)

Group	Dosage (mg/kg)	CD4^+^	CD8^+^	CD4^+^/CD8^+^
Model control	—	34.59 ± 3.07	20.64 ± 1.43	1.68 ± 0.14
5-FU group	20	22.86 ± 1.35*	17.37 ± 1.09	1.32 ± 0.31*
CEPS group	50	34.64 ± 2.89	20.67 ± 0.84	1.68 ± 0.17
	100	35.22 ± 2.42	20.89 ± 1.78	1.69 ± 0.20
	200	35.73 ± 3.77*	21.03 ± 1.24*	1.70 ± 0.34*
Se-CEPS group	50	35.04 ± 3.13	20.81 ± 1.17	1.68 ± 0.27
	100	37.38 ± 2.962*	21.39 ± 0.92*	1.75 ± 0.21*
	200	43.46 ± 4.83*	21.76 ± 1.58*	2.00 ± 0.28*
Se-CEPS + 5-FU group	200(Se-CEPS) + 20(5-FU)	31.25 ± 2.71*#	19.23 ± 1.30*#	1.63 ± 0.19*#

In the tumor-bearing mouse model, Se-CEPS intragastric administration significantly improved the immune organ index values and TNF-α and IL-2 levels in the serum, increased the proportion of cytotoxic T lymphocytes, and inhibited the growth of the transplanted tumors. In addition, Se-CEPS enhanced the anti-tumor activity of 5-FU and reduced the damage to the immune organs of the mice. 5-FU is an important chemotherapy drug for cancer treatment; however, the drug can cause severe bone marrow suppression, infection, hair loss, vomiting, and other serious side effects, and it can even endanger the patient’s life. The combination of polysaccharide and chemotherapy can improve the antitumor activity of chemotherapeutic drugs and reduce their side effects [21].

The body’s immune function, including the two aspects of cellular immunity and humoral immunity, is closely related to the occurrence and development of tumors. In particular, cell immunity has a primary role in tumor clearance [22]. Tumors are known to induce the production of inhibitory T cells and inhibitory macrophages, which can inhibit the production of IL-2 lymphocytes and promote tumor growth [23, 24]. To a certain extent, IL-2 activity reflects the body’s immune monitoring function, and its main biological activity involves promoting the proliferation of T lymphocytes and NK cells, differentiation and proliferation of B cells, and production of antibodies, among other functions [25]. Therefore, IL-2 has an important role in anti-tumor immunity [26]. IL-2 is an important immune regulatory protein that positively promotes a variety of immune cell activities [27]. For example, IL-2 can induce the differentiation of cytotoxic T lymphocytes and lymphokine-activated killer cells [28], which are both crucial in killing tumor cells [29]. The cytokine TNF-α has a variety of biological activities [30]. This cytokine can directly kill tumor cells, induce the apoptosis of tumor cells, and participate in the resistance to infection by bacteria, viruses, and parasites. Moreover, TNF-α can induce cell differentiation and promote mononuclear cells or T cells to secrete a variety of cytokines [31]. Mature T lymphocytes can be divided into two subsets: CD4^+^ and CD8^+^ [32, 33]. CD4^+^ cells are T helper cells that aid in secreting numerous cytokines and enhance the killing effect of CD8^+^ cells in tumors [34]. CD8^+^ cells are cytotoxic and suppressor T cells and act as important effector cells [35]. The lack of CD8^+^ and CD4^+^ T lymphocytes can lead to a weak immune response against tumors [36]. In general, our results demonstrate that Se-CEPS not only inhibits the formation of CT26 colon carcinoma but also improves the levels of IL-2 and TNF-α in the serum of mice. The results show that the effects of Se-CEPS on tumor inhibition and cell immune function and the secretion of IL-2 and TNF-α in the CT26 colon cancer mouse model are relevant. The level of CD8^+^ in the spleen of tumor-bearing mice was significantly increased by Se-CEPS treatment, indicating that Se-CEPS promotes the proliferation and activation of CD8^+^ T lymphocytes, directly kills tumor cells, and exerts an anti-tumor effect. Radiotherapy and chemotherapy are the most commonly used treatments for tumors, but they cause the inhibition of immune function and other serious side effects. The ability of Se-CEPS to enhance immune activity indicates its considerable potential for use in the treatment of tumors in the future.

## Conclusions

The results demonstrate the optimal fermentation conditions for the production of extracellular selenylated polysaccharide from *C. sobolifera* mycelium. The ultraviolet and infrared spectral analyses showed that selenium was successfully enriched in the extracellular polysaccharide. Moreover, activity tests showed that Se-CEPS improved the immune organ index of CT26 tumor-bearing mice and increased the TNF-α and IL-2 levels in serum. In addition, Se-CEPS improved the proportions of CD8^+^ and CD4^+^ T lymphocytes in the spleen, thereby inhibiting tumor growth. When combined with 5-FU, Se-CEPS improved the antitumor activity and reduced the side effects of the drug.

## Methods

### Material

*C. sobolifera* was collected and isolated from the Zhejiang Anji Bamboo Garden. CT26 colon carcinoma (CT26) cells were obtained from the American Type Culture Collection (ATCC, Manassas, VA). The 5-fluorouracil (5-FU) were purchased from Sigma (USA). Mouse ELISA kits for Interleukin 2 (IL-2) and tumor necrosis factor-α (TNF-α) were supplied by 4A Biotech Co. Ltd. (China). Fluorescein isothiocyanate (FITC)-conjugated anti-Mouse CD4 and phycoerythrin (PE)-conjugated anti-Mouse CD8 monoclonal antibodies were provided by eBioscience (USA). All other chemicals and reagents used were of analytical grade.

### Culture medium

The seed medium contained the following: potato extract, 20 %; glucose, 4 %; peptone, 0.2 %; yeast extract, 0.2 %; KH_2_PO_4_, 0.1 %; and MgSO_4_, 0.05 %. It was sterilized at 121 °C for 20 min.

The submerged fermentation medium contained the following: potato extract, 20 %; glucose, 4 %; peptone, 0.4 %; MgSO_4_, 0.05 %; KH_2_PO_4_, 0.1 %; yeast extract, 0.2 %; and vitamin B1, 0.002 %. It was sterilized at 121 °C for 20 min.

### Experimental method

#### Preparation and culture of the seed

A small activation inoculum was inoculated into a 250 mL Erlenmeyer flask. The volume was 100 mL, and it was shaken and cultured for at 26 °C for 3 d at 160 rpm. An 8 % (v/w) inoculum was transferred to 50 mL of sterilized fermentation medium in a 250 mL flask and then cultured for 120 h under the same conditions. The content of extracellular polysaccharide was measured in the fermentation broth after centrifugation at 4000 rpm for 15 min. All treatments were conducted three times.

### Extraction of extracellular polysaccharides

After centrifugation of the fermentation broth at 4000 rpm for 15 min, the supernatant was obtained. The supernatant was mixed with three volumes of 95 % ethanol (v/v), stirred vigorously, and left overnight at 4 °C. The precipitated polysaccharides were centrifuged at 8,000 × *g* for 15 min, and the supernatant was discarded. The polysaccharide precipitates were washed three times with 70 % ethanol and lyophilized to a constant weight in vacuo.

### Optimization of the fermentation medium

Potato juice (20–60 %), peptone (0.2–1.0 %), and KH_2_PO_4_ (0.2–0.6 %) were examined using a three-factor and three-level L_9_ (3^3^) orthogonal array design. In addition, analysis of variance (ANOVA) was used to evaluate the statistical significance of the effects of the individual factors on Se-CEPS production. The yield of Se-CEPS was further evaluated to confirm the production rate of target compounds based on the optimized conditions.

### Construction of the tumor bearing mouse model

After CT26 cells were grown to an 80 to 90 % fusion rate, the cells were digested in trypsin and collected. The cell concentration was adjusted to 5 × 10^6^/mL using phosphate-buffered saline (PBS). Seventy-two BALB/c mice were used in the study. The healthy mice were purchased from the Laboratory Animal Center of Zhejiang University (*Institutional Animal Welfare and Ethics Committee* of Zhejiang University, China). This study was approved by the Animal Ethics Committee of Zhejiang University. A 75 % alcohol solution was used to disinfect the armpit of the right arm of the mice, and a 0.2 mL CT26 cell suspension was then inoculated subcutaneously. On the seventh day after the inoculation of tumor cells and after a 80–90 mm^3^ tumor had formed at the inoculation site, the mice were randomly divided into nine groups: the control group (saline group), Se-CEPS or CEPS high dose group (200 mg/kg/d), Se-CEPS or CEPS middle dose group (100 mg/kg/d), Se-CEPS or CEPS low dose group (50 mg/kg/d), Se-CEPS + 5-FU group (200 mg/kg/d Se-CEPS + 20 mg/kg/d 5-FU), and positive control group (5-FU: 20 mg/kg/d). The intragastric administration of the drug was performed once a day for 14 d.

### Analysis method

#### Measurement of polysaccharides in the fermentation broth

The extracellular polysaccharides were measured using the phenol sulfuric acid method [37].

### Measurement of selenium content

Selenium was measured in selenium-enriched polysaccharides using a PerkinElmer AAanalvst800 atomic absorption spectrophotometer.

### Ultraviolet spectrum analysis of Se-CEPS and CEPS

Ten percent Se-CEPS and CEPS solutions were prepared using ultrapure water as a blank control, and ultraviolet scanning analysis was conducted in the range of 200–400 nm.

### Infrared spectrum analysis of Se-CEPS and CEPS

The infrared (IR) spectra were recorded using the KBr-disc method with a Fourier transform infrared (FTIR) spectrometer (Tensor27 Fourier transform infrared spectrometer; Bruker, Germany) in the range of 400–4,000 cm^−1^.

### Measurement of the tumor inhibition rate, spleen index, and thymus index

On the 15th day after drug administration, the tumor-bearing mice were weighed. The eyeballs were removed, blood was extracted, and animals were sacrificed through cervical dislocation. The tumor, spleen, and thymus from each animal were isolated and weighed. The tumor inhibition rate, spleen index, and thymus index were calculated. The blood samples were stored at room temperature. After coagulation and centrifugation at 3000 rpm for 10 min, the mouse serum was added to Torikami Kiyo medium, which was used for the determination of cytokines.

$$ Tumor\  Inhibition\  Rate=\left( Average\  tumor\  weight\  of\  the\  model\  control\  group\hbox{--} Average\  tumor\  weight\  of\  the\  experimental\  group\right)/ Average\  tumor\  weight\  of\  the\  model\  control\  group\times 100\;\% $$

$$ Spleen\  Index\left( mg/g\right)= Average\  spleen\  weight/ Average\  body\  weight $$

$$ Thymus\  Index\left( mg/g\right)= Average\  thymus\  weight/ Average\  body\  weight $$

### Determination of serum cytokine levels by the ELISA assay

The levels of IL-2 and TNF-α in the serum of the mice from each group were determined using commercial mouse ELISA kits according to the manufacture’s protocols.

### CD8^+^ T and CD4^+^ T lymphocyte analysis

Mice were killed by cervical dislocation, and their spleens were removed. One milliliter PBS was added to each spleen, and the spleen was teased apart into a single cell suspension by passing it through a 3 mL syringe. The suspension was subjected to red blood cell lysis and 1000 rpm centrifugation for 10 min and then washed with sterile PBS once. The suspension was combined with 1.5 μL FITC-CD3, 3.75 μL anti-CD8, or anti-CD4 and then incubated in the dark at 4 °C for 30 min. A 0.05 % sodium azide PBS solution was used to wash away the unbound antibody, and the cells were resuspended in 200 μL 2.0 fetal bovine serum and 0.05 % sodium azide PBS buffer. BD FACSVerse flow cytometry was performed, and the data were obtained and analyzed using FACSuite software.

### Statistics processing

All data are expressed as the mean ± standard error. SPSS17.0 statistical software (SPSS Inc. 233 South Wacker Drive, 11th Floor, Chicago) was used for single factor analysis of variance (one-way ANOVA). A *p* value < 0.05 indicates a statistically significant difference.

## Abbreviations

5-FU, 5-fluoro-2,4(1 h, 3 h)pyrimidinedione; ANOVA, analysis of variance; *C. sobolifera*, extracellular polysaccharide; *C. sobolifera*, *Cordyceps sobolifera*; CD4^+^, Cluster of Differentiation 4; CD8^+^, Cluster of Differentiation 8; CT26, Colonic adenocarcinoma cell; ELISA, Enzyme-linked immuno sorbent assay; IL-2, Interleukin-2; IR, infrared; CEPS, *C. sobolifera* extracellular polysaccharide; PBS, phosphate-buffered saline; Se-CEPS, selenium-enriched; TNF-α, Tumor Necrosis Factor; UV, Ultraviolet

## Additional file

Additional file 1: Fermentation medium optimization of Cordyceps sobolifera extracellular polysaccharide (CEPS). **Figure S1.** Effects of carbon source on CEPS. **Figure S2.** Effects of concentration of potato on CEPS. **Figure S3.** Effects of nitrogen source on CEPS. **Figure S4.** Effects of concentration of peptone on CEPS. **Figure S5.** Effects of inorganic salt on CEPS. **Figure S6.** Effects of concentration of KH2PO4 on CEPS. (DOC 29 kb)
