# Supplementary material for: Optimization of the fermentation process of Cordyceps sobolifera Se-CEPS and its anti-tumor activity in vivo
Source: J Biol Eng. 2016 Jun 23;10:8. doi: 10.1186/s13036-016-0029-0 (PMC4919858; doi:10.1186/s13036-016-0029-0)
Supplement: Additional file 1: — Fermentation medium optimization of Cordyceps sobolifera extracellular polysaccharide (CEPS). Figure S1. Effects of carbon source on CEPS. Figure S2. Effects of concentration of potato on CEPS. Figure S3. Effects of nitrogen source on CEPS. Figure S4. Effects of concentration of peptone on CEPS. Figure S5. Effects of inorganic salt on CEPS. Figure S6. Effects of concentration of KH2PO4 on CEPS. (DOC 29 kb) [file 13036_2016_29_MOESM1_ESM.doc]

### Fermentation medium optimization of *Cordyceps sobolifera* extracellular polysaccharide (CEPS)

The effect of the substrate concentration in the medium on *Cordyceps sobolifera* extracellular polysaccharide (CEPS) production was studied.

Carbon source was added to the culture medium, and the effect of carbon source on CEPS production is shown in Fig. S1. Potato juice was the most efficient carbon source, which resulted in the highest CEPS content. From Fig. S2, it is seen that the CEPS yield increased with the concentration of potato juice and reached maxima at 20%(v/v).

Various nitrogen sources, at mass concentration of 1.0 %, were added separately to the medium containing 20 % potato juice. The results are shown in Fig. S3. Peptone was the most efficient nitrogen source, which resulted in the highest CEPS content. The others were followed by beef extract, yeast extract, ammonium sulfate, urea, soybean cake, and bran, respectively. From Fig. S4, it is seen that the CEPS yield increased with the concentration of peptone and reached maxima at 0.6%(m/v).

In order to obtain a higher product yield, optimization of the inorganic salt composition in the medium is also important besides the fundamental medium composition, as me ntioned in the above subsection. KH_2_PO_4_, K_2_HPO_4_, NaCl, CaCl_2_, MgSO_4_, FeSO_4_ are the major inorganic constituents of fungi; these minerals should be supplied sufficiently, and optimization of the additional concentration is important. Therefore, various groups have attempted to enhance CEPS productivity by optimization of the amounts of inorganic salt added. KH_2_PO_4_ was the most efficient inorganic salt, which resulted in the highest CEPS content in Fig. S5. From Fig. S6, it is seen that the CEPS yield increased with the concentration of KH_2_PO_4_ and reached maxima at 0.4%(m/v).

**We (all authors) have not Competing Interest section header.**

Fig.S1 Effects of carbon source on CEPS

Fig.S2 Effects of concentration of potato on CEPS

Fig.S3 Effects of nitrogen source on CEPS

Fig.S4 Effects of concentration of peptone on CEPS

Fig.S5 Effects of inorganic salt on CEPS

Fig.S6 Effects of concentration of KH_2_PO_4_ on CEPS
